# Trisomy 21 with Maternally Inherited Balanced Translocation (15q;22q) in a Female Fetus: A Rare Case of Probable Interchromosomal Effect

**DOI:** 10.3390/cells13131078

**Published:** 2024-06-21

**Authors:** Alessandro De Falco, Antonella Gambale, Michele Pinelli, Teresa Suero, Luigia De Falco, Achille Iolascon, Stefania Martone

**Affiliations:** 1 U.O.C. Genetica Medica, A.O.U. Federico II, 80131 Naples, Italy; ale.deltafi@gmail.com (A.D.F.); antonellagambale@gmail.com (A.G.); michele.pinelli@unina.it (M.P.); achille.iolascon@unina.it (A.I.); 24.stefania.martone@gmail.com (S.M.); 2 Department of Molecular Medicine and Medical Biotechnology, University Federico II, 80131 Naples, Italy; 3 AMES-Centro Polidiagnostico Strumentale, Srl, 80013 Naples, Italy; teresasuero83@gmail.com; 4 CEINGE Biotecnologie Avanzate, 80145 Naples, Italy

**Keywords:** Robertsonian translocation, trisomy 21 (Down syndrome), interchromosomal effect (ICE) prenatal genetic counseling, karyotype, SNP array

## Abstract

Chromosomal rearrangements can interfere with the disjunction and segregation of other chromosome pairs not involved in the rearrangements, promoting the occurrence of numerical abnormalities in resulting gametes and predisposition to trisomy in offspring. This phenomenon of interference is known as the interchromosomal effect (ICE). Here we report a prenatal case potentially generated by ICE. The first-trimester screening ultrasound of the pregnant woman was normal, but the NIPT indicated a high risk for three copies of chromosome 21, thus suspecting trisomy 21 (T21). After a comprehensive clinical evaluation and genetic counseling, the couple decided to undergo amniocentesis. The prenatal karyotype confirmed T21 but also showed a balanced translocation between the long arm of chromosome 15 (q22) and the long arm of chromosome 22. The parents’ karyotypes also showed that the mother had the 15;22 translocation. We reviewed T21 screening methods, and we performed a literature review on ICE, a generally overlooked phenomenon. We observed that ours is the first report of a prenatal case potentially due to ICE derived from the mother. The recurrence risk of aneuploidy in the offspring of translocated individuals is likely slightly increased, but it is not possible to estimate to what extent. In addition to supporting observations, there are still open questions such as, how frequent is ICE? How much is the aneuploidy risk altered by ICE?

## 1. Introduction

Robertsonian translocations (RTs) involve the fusion of two acrocentric chromosomes, typically chromosomes 13, 14, 15, 21, or 22, resulting in the loss of parts of their short arms during subsequent cell divisions. They are the most common type of chromosome rearrangement, occurring at a rate of approximately 1 in 800 in the general population [1]. RTs occur predominantly during female meiosis and are classified into two classes: Class I (e.g., rob(13;14) and rob(14;21)) which result from recombination between the same breakpoints (microsatellite arrays) occurring mostly during oogenesis, and Class II (all the others) which have heterogeneous breakpoints and variable losses [2]. RT carriers are typically assumed to exhibit no phenotypic abnormalities but are at increased risk for reproductive challenges like miscarriages, infertility, uniparental disomy, and aneuploid offspring due to the production of unbalanced gametes [3].

However, recent evidence suggests that RTs may have phenotypic effects beyond reproductive issues, including elevated risks for breast cancer or hematologic malignancies [4]. This challenges the traditional view and underscores the need to reconsider the potential pathogenic mechanisms of RTs, as well as explore other ways in which structural genome variations may impact phenotype.

Trisomy 21 (T21) is a genetic disorder caused by the presence of an extra chromosome 21 and it manifests as a diverse pattern of congenital malformations and dysmorphisms. T21 is a common cause of intellectual disability with an estimated prevalence of 9.2 cases per 10,000 live births in Europe. Without elective terminations, live birth prevalence would have been around 21.7 per 10,000 live births, or 17,331 births annually [5]. The likelihood of having a child with Down syndrome follows a steady, linear progression until approximately age 30, after which it escalates exponentially [6]. At 25 years old, the risk of having a child with T21 is 1 in 1300 for women. By age 35, this risk rises to 1 in 365, and by age 45, the likelihood of having a child with T21 surges to 1 in 30 [7].

If all pregnant women aged 35 years or older opted for amniocentesis, approximately 30 percent of pregnancies with T21 would be identified [8]. Conversely, women younger than 35 years account for around 70 percent of T21 cases [9]. Maternal serum screening, also known as multiple-marker screening, enables the detection of T21 pregnancies in this younger demographic.

The triple test, combining alpha-fetoprotein (AFP), unconjugated estriol, and human chorionic gonadotropin (hCG), serves as the primary screening method [10]. AFP originates from the yolk sac and fetal liver, while unconjugated estriol and hCG derive from the placenta. During T21 pregnancies, second-trimester maternal serum levels of AFP and unconjugated estriol decrease by about 25 percent, while maternal serum hCG levels rise to approximately double the normal level [9].

Typically administered between 15 to 18 weeks of gestation, the triple test measures each serum marker relative to the median for pregnancies of similar gestational age (multiple of the median, MoM). The risk of T21 is then calculated based on these results and the patient’s age, with a standard risk cutoff of 1/270 commonly utilized [10]. A positive test prompts chorionic villus sampling or amniocentesis [11]. The triple test identifies 60% of T21 pregnancies with a 5 percent false positive rate. A normal result decreases the likelihood of T21 but does not rule it out completely. Adjustments for maternal factors such as weight, ethnic background, and insulin-dependent diabetes mellitus can slightly enhance test accuracy [9].

During the first trimester, the ultrasound measurement of nuchal translucency combined with the maternal age, it is possible to achieve a T21 detection rate of 63 percent, with a false positive rate of 5% [12]. Combining this approach with the measurement of maternal serum-free beta-hCG subunit and pregnancy-associated protein A (PAP A) could raise the detection rate to 80% while maintaining the same false positive rate [13].

The non-invasive prenatal testing (NIPT) emerges as a milestone in the history of prenatal diagnosis, offering an advanced and non-invasive approach to identifying chromosomal abnormalities in the fetus. This technology, based on the detection of fetal DNA in maternal blood, represents a significant evolution compared to traditional screening methods during pregnancy. The presence of fetal DNA in maternal serum was first observed in 1997 [14] when fetal-derived Y chromosome sequences were detected in 80% of plasma and maternal serum samples from women with male fetuses, and no positive samples were found in 13 women with female fetuses and 10 non-pregnant women.

Circulating cell-free fetal DNA (cffDNA) derives from trophoblast cell apoptosis and is therefore of placental origin [15,16]. It has a short half-life [14], is cleared from maternal circulation, likely through renal excretion, and is not detectable after two hours postpartum [17]. This means there is no memory of previous pregnancies, although there could be a possible interference by cfDNA from a vanished twin [18].

Confirmation of an ongoing pregnancy via ultrasound and determination of parity are essential before discussing and planning any pregnancy tests. Accurate ultrasound dating of the pregnancy is crucial to increase the likelihood of sufficient cffDNA in maternal blood necessary for NIPT testing. Additionally, ultrasound evidence of structural abnormalities may indicate the need to forego screening and opt for invasive prenatal diagnosis (chorionic villus sampling or amniocentesis) [19].

In Italy, NIPT is recommended as a screening test for common chromosomal aneuploidies (T21, T18, T13), which represent about 50% of clinically relevant fetal chromosomal abnormalities [20].

The advantages over other non-invasive screening tests include higher detection rates, higher positive and negative predictive values (especially for T21 and T18), and less dependence on gestational age. It can be performed from the 11th week of gestation until the end of pregnancy [21] (Table 1).

The testing method that provides the earliest and most precise detection of T21, is also NIPT for the early detection from the 11th week of pregnancy, for its non-invasiveness and accuracy especially if combined with the first-trimester ultrasound.

In cases of a previous T21 pregnancy, the risk of recurrence of a chromosomal anomaly in subsequent pregnancies rises by approximately 1% above the baseline risk determined by maternal age [22,23]. If a chromosome-21 translocation is diagnosed in the fetus or newborn, it prompts karyotype analysis of both parents. When both parents exhibit normal karyotypes, the recurrence risk falls within the range of 2 to 3%. However, if one parent carries a balanced translocation, the recurrence risk is influenced by the sex of the carrier parent and the specific chromosomes involved [22]. Women who carry an RT involving chromosome 21 generally have a higher recurrence risk of approximately 10–15% of having a child with T21 compared to male carriers. Men who carry an RT involving chromosome 21 have a significantly lower recurrence risk compared to female carriers: the recurrence risk is usually less than 1–3% [24]. The difference in recurrence risk is largely due to the way gametes are formed. In female carriers, there is a higher likelihood that an egg with an unbalanced chromosomal composition will be viable and result in a live birth [25]. In male carriers, instead, many of the sperm with unbalanced chromosomal compositions are less likely to be viable or capable of fertilization [26]. Moreover, female meiosis and the mechanisms involved in oocyte selection tend to tolerate chromosomal imbalances differently than male meiosis and spermatogenesis. This contributes to the higher risk observed in female carriers [24].

The significance of a family history of T21 varies based on the karyotype of the affected individual (proband). If the proband has T21, the likelihood of a T21 pregnancy remains only slightly increased for other family members besides the parents. However, if the proband has a chromosome-21 translocation or if the karyotype is unknown, family members should be offered genetic counseling and karyotype analysis [27].

There are other conditions suspected to increase the risk of T21 recurrence: parental mosaicism for T21 [28], a parent carrying a balanced translocation [24], the age of the mother at conception [29], previous child with T21, and other genetic syndromes that affect chromosomal stability, like Bloom syndrome, increasing the risk of chromosomal nondisjunctions leading to conditions like T21 [30].

The occurrence of T21 alongside a reciprocal translocation between chromosomes other than 21 in the fetal karyotype structure is an uncommon phenomenon, sparsely documented in the literature [31,32,33]. The occurrence of RT can interfere with the disjunction and segregation of other chromosome pairs not involved in the rearrangements, like chromosome 21, promoting the occurrence of numerical abnormalities in resulting gametes. This interference phenomenon is known as the interchromosomal effect (ICE). In fact, in 1963, Lejeune observed an increased frequency of reciprocal translocation carriers among the fathers of children with T21 and he was the first scientist to propose the existence of the ICE [31,32,34].

Here, we describe a prenatal case with both T21 and balanced translocation between the long arm of chromosome 15 (q22) and the long arm of chromosome 22 (q13.1). In particular, we reviewed the current literature on ICE and identified the elements that led us to suspect its occurrence in our case. We further speculated on how knowledge of the existence of ICE would affect genetic prenatal counseling for cases like ours.

### Clinical Description

**Figure 1:**
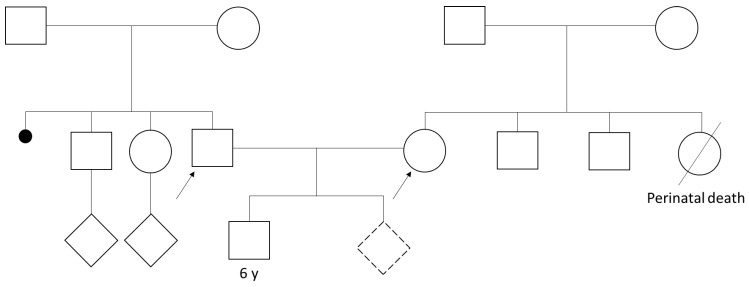
Pedigree of the presented couple. The dotted rhombus is the proband fetus of the couple. The signs represent the following: white circles—female, white squares—male, rhombus—unknown sex, dotted rhombus—unknown sex in fetus, diagonal line—dead person, little solid circle—abortion, straight line connecting two symbol-marriage, the arrows identify the two members of the presented couple.

Regarding the family history of our prenatal genetic counseling, no major pathologies emerged. The male consultand (33 years) reports that his mother had an abortion in the first trimester of pregnancy. The pregnant woman (37 years) reports that a sister died a few hours after birth due to unspecified problems (Figure 1). Consanguinity between the partners was denied.

Since our genetic unit is a center of reference for inherited blood disorders and thalassemia, widely present in our country, we require a complete blood count (CBC) of the consultands at every genetic consultation. According to the clinical documentation, the CBCs and the hemoglobin electrophoresis of both consultands were normal, and at low risk for thalassemia (Table 2).

Regarding the gestational history, the first trimester screening ultrasound was normal: particularly, the nuchal translucency (1.39 mm) and the fetal anatomy were both normal.

The pregnant women decided to perform NIPT, which showed a high risk for trisomy 21. After a comprehensive evaluation during the prenatal genetic counseling, we have discussed how NIPT is a screening test and not a definitive diagnosis. We have also discussed the opportunity of further diagnostic testing such as the chorionic villus sampling or the amniocentesis. The couple decided to undergo amniocentesis.

## 2. Materials and Methods

### 2.1. Molecular Cytogenetic Analysis

For NIPT analysis, a total of 10 mL peripheral blood was collected at 12 + 0 weeks and NIPT was carried out using VeriSeqTM NIPT Solution v2 assay (Illumina Inc., San Diego, CA, USA) [35,36]. The pregnant woman chose the genome-wide analysis report, used to receive results about common trisomies (13, 18, and 21) and sex chromosomes, as well as results on rare autosomal aneuploidies and partial deletions/duplications ≥ 7 Mb. Fetal fraction was 14%.

Genomic DNA was extracted from peripheral blood in vacutainers with EDTA using MagCore Nucleic Acid Extraction Kit according to the manufacturer’s instructions (Diatech Pharmacogenetics, Jesi, Italy). DNA quantity and purity were determined with NanoDrop One (ThermoScientific, Waltham, MA, USA).

Amniotic fluid was drawn, and genomic DNA was extracted from the amniocyte using the QIAamp DNA Blood Mini Kit (Qiagen, Hilden, Germany). The quantitative fluorescent polymerase chain reaction test for rapid aneuploidy detection was performed using the Devyser Compactv3 QF-PCR Kit (QF-PCR; Devyser Compactv3, Devyser, Stockholm, Sweden) as previously described [37]. PCR products were separated by electrophoresis using an ABI 3130xl Genetic Analyzer, and the analysis of each allele for specific markers was performed using GeneMapper Software ver. 4.0 (Applied Biosystems, Waltham, MA, USA). GTG-banding analysis of amniotic fluid was performed in established cell cultures following standard laboratory protocols. Forty metaphases were analyzed with the CytoVision software (CytoVision Version 7.6, Leica Biosystems, Richmond, IL, USA).

SNP array analysis was performed using a HumanCytoSNP-12 v2.1 kit, following the manufacturer’s protocol (https://emea.support.illumina.com/content/dam/illumina-support/documents, accessed on 1 December 2022). Stained bead chips were scanned with a HiScan™SQ System (Illumina, Inc., San Diego, CA, USA). Data were generated with GenomeStudio software 2.0 (Illumina, Inc., San Diego, CA, USA) and analyzed with the Bluefuse Multi Software v4.5 (Illumina, Inc., San Diego, CA, USA). All CNVs > 100 Kb were interrogated. All results were reported according to the GRCh37 (hg19) assembly.

### 2.2. Ethical Consent

Clinical investigations and genetic analyses were conducted in accordance with the principles of the Declaration of Helsinki. Ethical review and approval were waived for this study, dealing with a case report conducted according to clinical practice guidelines.

Written informed consent has been approved by both the consultands.

## 3. Results

**Figure 2:**
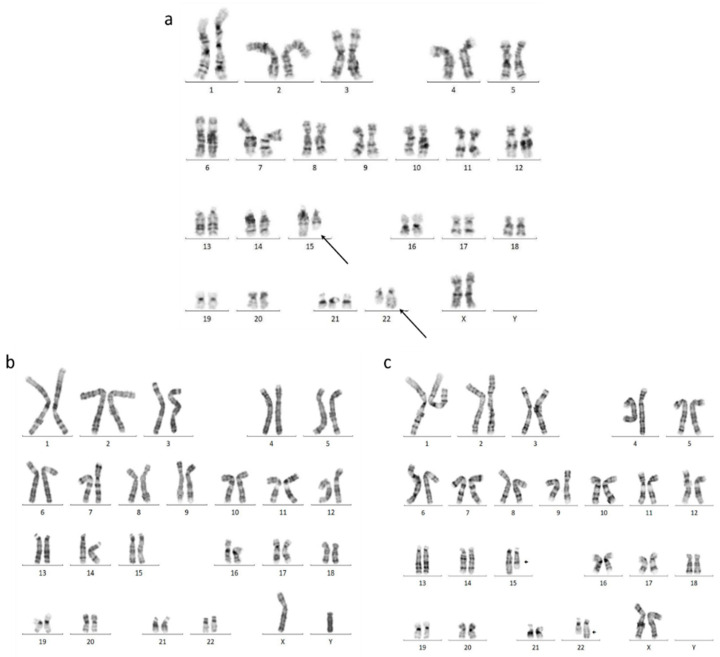
Karyotypes. (**a**) Karyotype, performed on cultured amniocytes: 47,XX,t(15;22)(q22;q13.1),+21. The arrows stands for the chromosome involved in the translocation. (**b**) Karyotype of the father: 46,XY. (**c**) Karyotype of the mother 46,XX,t(15;22)(q22;q13.1).

The karyotype, performed on cultured amniocytes, showed the following result: 47,XX,t(15;22)(q22;q13.1),+21 standing for a trisomy of chromosome 21 and a balanced translocation between the long arm of a chromosome from the pair 15 (q22) and the long arm of a chromosome from the pair 22(q13.1) (Figure 2a).

We studied the karyotype from peripheral blood in the parents. The father’s karyotype was normal (46, XY) (Figure 2b) while the mother’s had the same translocation as the fetus (46,XX,t(15;22)(q22;q13.1)) (Figure 2c).

**Figure 3:**
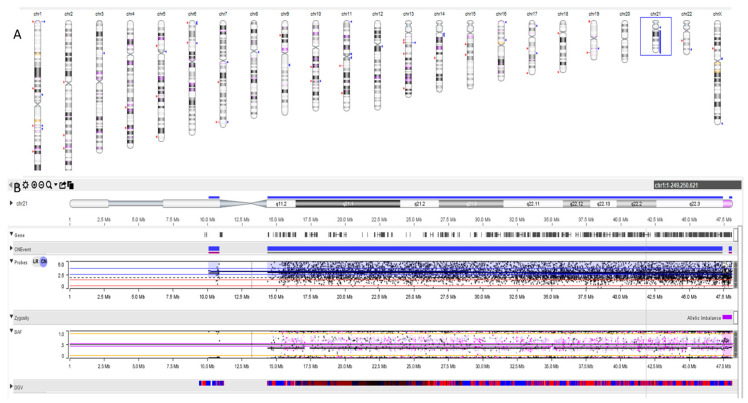
Results of SNP array analysis performed on amniotic fluid. (**A**) Ideogram of SNP array analysis of amniotic fluid showed a duplication (in blue) of chromosome 21 in the fetus’s amniocytes. (**B**) Upper panel. Ideogram of chromosome 21 of the proband: detailed depiction of the duplication of chromosome 21. Bottom panel. SNP distribution. LRR is the logged ratio of observed probe intensity to expected intensity: so, any deviations from zero in this metric are the evidence for copy number changes. Black dots above red and pink lines indicate copy number changes for a region. BAF is the proportion of hybridized sample that carries the B allele as designated by the Infinium assay. In a sample with a normal karyotype, discrete BAFs of 0.0, 0.5, and 1.0 are expected for each locus (representing AA, AB, and BB). In trisomy, we have 4 populations: AAA, AAB, ABB, and BBB. Scattered dots between orange lines indicate trisomic representations of alleles.

We also performed an SNP array in trio on cultured amniocytes and cultured leucocytes from the peripheral blood of each parent: we found that the additional chromosome 21 found in the fetal karyotype derives from the mother as well as the translocated chromosome t(15;22)(q22;q13.1) chromosome (Figure 3).

## 4. Discussion

Meiosis is a highly regulated process that involves a first stage (prophase I) where homologous recognition between chromosome pairs is required to allow their complete pairing (homosynapsis). The presence of chromosomal variants, like RT, causes significant challenges to both identifying homology and distributing crossovers [38,39], potentially compromising the proper progression of gametogenesis in the affected carriers and also the fertility through two fundamental mechanisms: (i) abnormal progression of meiosis with complex pairing geometries due to the formation of synaptic regions leading to low production of gametes. In fact, during Prophase I, rearranged chromosomes adopt complex pairing geometries, often leading to the formation of asynaptic regions near breakpoints [40]. This disruption hampers the achievement of homosynapsis at several cycle checkpoints, resulting in reduced gamete production. (ii) Generation of unbalanced gametes: carriers of structural reorganizations may experience fertility issues due to the production of gametes with chromosomal imbalances [38]. These imbalances arise primarily from various modes of unbalanced segregation of rearranged chromosomes. Moreover, such rearrangements can interfere with the disjunction and segregation of other chromosome pairs not involved in the rearrangements, promoting the occurrence of numerical abnormalities in resulting gametes and predisposition to trisomy offspring [34].

**Figure 4:**
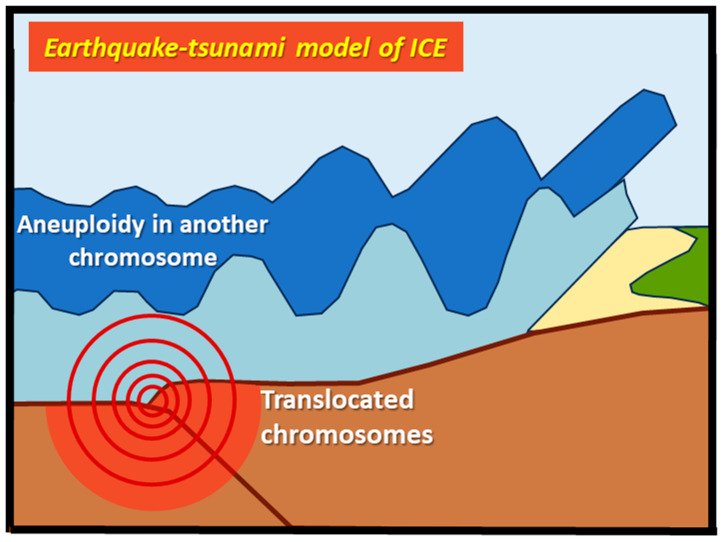
Representation of ICE in earthquake–tsunami analogy. Like an earthquake that creates a tsunami, the ICE is the phenomenon for which translocated chromosomes (which can be compared to the epicenter of an underwater earthquake) interfere with the disjunction and segregation of other chromosome pairs not involved in the rearrangements (which can be compared to the tsunami generated by the earthquake). It is plausible to imagine that a different “geography” of the chromosomes within the nucleus (the epicenter of the earthquake), imposed by the complicated synapsis of the translocated chromosomes, could perturb the distribution of other “bystander” normal chromosomes at meiosis (like a tsunami), including chromosome 21.

This interference phenomenon is known as the interchromosomal effect (ICE). ICE has been implicated in cases where standard T21 occurs alongside a parental karyotypic abnormality unrelated to chromosome 21 (as an RT between chromosomes 13 and 14, or a reciprocal translocation). The complex synapsis of these translocated chromosomes within the nucleus might create a different chromosomal “landscape”, potentially impairing the balanced distribution of the other “bystander” normal chromosomes during meiosis, including chromosome 21 [34]. We propose the following model of ICE, we call it the earthquake–tsunami effect (Figure 4).

The onset of ICE is intricately linked to the development of heterosynapses among the meiotic configurations formed by the chromosomes undergoing reorganization. These chromosomes typically exhibit regions where synapses fail to develop, alongside other sensitive areas within the genome [40,41,42]. Certain genomic regions display a heightened inclination to participate in these irregular synapses. These regions may either exhibit a greater propensity toward heterosynapsis or harbor gaps or breaks within the synaptonemal complex [43].

Furthermore, it is well known that chromosomal territories, which are distinct, non-overlapping regions of the nucleus occupied by individual chromosomes, are important for various cellular processes, including transcription and DNA repair. In the context of chromosomal territories, the spatial organization of chromosomes within the nucleus can influence gene regulation and chromosomal interactions. In particular, replication time is the temporal order in which different regions of the genome are replicated during the S phase of the cell cycle [44]. Chromosomes and their territories can have distinct replication timing profiles, and changes in replication timing can reflect changes in chromosomal organization. Cremer and Branco have demonstrated that acrocentric chromosomes, including chromosomes 15, 21, and 22, are frequently positioned near each other. This spatial proximity within the nucleus can facilitate interchromosomal interactions, which may be relevant to both functional interactions and regulatory mechanisms of chromosomes, including replication time and ICE [44,45].

Enlightening studies have established a correlation between the occurrence of these interchromosomal heterosynaptic interactions and phenomena like meiosis [46,47]. They have demonstrated how such events coincide with alterations in seminal parameters, a characteristic that tends to accumulate in individuals carrying structural chromosome rearrangements [48]. However, an alternative hypothesis suggests that the occurrence of heterosynapses might serve as a cellular mechanism designed to overcome and so to rescue the meiotic arrest that would otherwise be caused by regions lacking synapses [49,50].

Alfarawati and colleagues have reported results from a collection of samples consisting of oocytes and embryos from translocation carriers. They collected 283 samples from chromosome rearrangement carriers and a control group of 5078 samples from karyotypically normal individuals. They made chromosome analyses of oocytes, cleavage stage embryos, and blastocysts using array CGH: 81.3% of samples from rearrangement carriers were chromosomally abnormal, compared to 65.3% in the control group. Excluding rearranged chromosomes, a significant increase in aneuploidy (0.051 vs. 0.047 of probability per chromosome) was observed in rearrangement carriers, suggesting an ICE. Detailed analysis of more than 210,000 chromosomes showed no significant ICE in reciprocal translocation carriers or inversion carriers, but a clear ICE was identified in RT carriers, particularly during the cleavage stage of embryo development. The ICE was not evident in oocytes or blastocysts, implying its effect during early mitotic divisions post-fertilization (three days after fertilization at the 6–10 cell stage). They concluded that RT carriers show an increased risk of aneuploidy due to an ICE during early mitotic divisions and this genetic instability could lead to a higher rate of miscarriage and reduced fertility [51].

The general idea coming from these studies is that the outcome of these interactions manifests as a significant increase in numerical chromosomal abnormalities within the resulting cells. This, in turn, contributes an additional source of chromosomal imbalance in carriers of such structural rearrangements. The perturbing effect of these interactions could be translated into a notable rise in numerical chromosome abnormalities in the resultant cells, further exacerbating chromosomal imbalance in carriers of reorganizations.

In 1963, Lejeune observed an increased frequency of reciprocal translocation carriers among the fathers of children with T21 and he was the first scientist to propose the existence of the ICE [32]. In documented cases, families have been identified where children exhibited isolated T21 alongside a balanced translocation impacting chromosomes other than 21, frequently of paternal origin [34]. In these cases, ICE increases the risk of T21 with the extra chromosome 21 deriving from the father who carries the translocated chromosome.

The question of whether ICE truly exerts an effect remains controversial. If such an effect does indeed exist, it seems to have infrequent practical consequences in most cases. Studies [31,33] have contributed to this ongoing debate. The ideal method to detect the existence of any ICE is the analysis of sperm cells before selection based on embryo viability. However, analyses of sperm in RT carriers have yielded conflicting outcomes.

In 2010, Anton, Vidal, and Blanco summarized data from 33 males carrying RT in a cohort from an infertility center. They performed sperm FISH studies finding that ICE was detected in the sperm of slightly more than half (20 out of 36) of the males studied. The main limitations of this study were the recruitment bias (men from an infertility center, as oligospermia is independently associated with heightened levels of aneuploidy in sperm) and also other aneuploidies were missed [31].

It has become clear that ICE is not a universal phenomenon and is observed only in some carriers of balanced chromosomal rearrangements, predominantly in patients with reproductive issues. In 2013, Kovaleva and colleagues determined whether ICE operates in the genome of fertile carriers of rearrangements, which specific rearrangements, and how significantly they increase the risk of aneuploidy. They used data on live-born children with Down syndrome born in Saint Petersburg from 1980 to 2010 which were obtained from the Saint Petersburg Down Syndrome Registry. A cytogenetic analysis of children with T21 and the parents of offspring with T21 was conducted using differential chromosome staining methods, such as FISH, Giemsa staining, and spectral karyotyping. Cases involving chromosome 21 rearrangements were excluded, and published data on newborns were used as controls, forming two control groups: one excluding de novo rearrangements (K1) for comparison with T21 patients, and one including de novo rearrangements (K2) for comparison with parents. The study identified 1796 children with T21 born between 1980 and 2010, with cytogenetic confirmation in 1496 cases. Among these, 83 (6%) had a translocation variant of T21, 50 (4%) had mosaic T21, and the remaining 1363 had free T21. Six cases of inherited chromosomal rearrangements were found in children with free T21. Frequencies of inherited, de novo rearrangements and combined frequency of inherited and de novo chromosomal rearrangements in K1 were 0.5, 0.8, and 0.2 per thousand, respectively, while in K2 were 0.9, 1.3, and 0.3 per thousand, respectively. These differences in frequencies suggest a higher baseline occurrence of chromosomal rearrangements in K2 compared to K1. This study found a significant prevalence of reciprocal translocations and inversions among parents of children with T21 compared to controls. The results suggest the influence of paternal rearrangements on maternal chromosome segregation post-fertilization. Unfortunately, the article is written in the Russian language, and not all of the text is fully comprehensible [33]. Despite the controversy, exploring the potential impact of ICE on chromosomal distribution during meiosis continues to be an area of active research and discussion within the scientific community.

## 5. Conclusions

Here, we have reported a prenatal genetic case of counseling concerning the occurrence of RT alongside T21 where the RT has maternal origin: this is the first case of possible ICE derived from the mother. We have conducted an SNP array, which allowed us to pinpoint unequivocally the origin of T21 and RT from the mother in a female fetus.

However, there are still open questions: does the ICE really exist? The available scientific evidence is not very strong. ICE is a hypothesis to explain the phenomenon observed in some cases of chromosomal trisomy. Maybe, there is a better term to describe the nondisjunction of a chromosome in the presence of a translocation if that chromosome itself is not involved in the translocation.

Given the real existence of this effect, what is the risk of recurrence of aneuploidies in a translocation carrier? Regarding our case report of prenatal genetic counseling, we have estimated a risk of recurrence of T21 in subsequent pregnancies in the couple by approximately 1:230 [52] related to the risk of maternal age augmented of 1% above the baseline risk determined by maternal age [22,23] for a total risk of about 1,43% of recurrence of T21.

Despite preliminary observations suggesting that ICE is an underestimated mechanism of aneuploidy generation, conclusive evidence is still lacking. We believe the main limitation is that both Robertsonian translocations and chromosomal aneuploidies are rare events, affecting only a small fraction of the general population. Consequently, their co-occurrence is an even rarer event, making it difficult to attribute it to either a causal association or random overlap. Analyzing sperm cell karyotypes from patients with translocations could provide the necessary statistical power to draw conclusions, at least for male meiosis. However, for females, as in the case we are studying, this uncertainty is likely to remain for a long time.

Additional carefully designed studies are needed to truly understand the impact of translocation on the frequency of nondisjunction in humans. Examining specific chromosomal interactions between chromosome 21 and other chromosomes (with Hi-C or 4C-seq) in sperm and ovarian cells could help to better understand the molecular mechanisms that happen inside the nucleus of these cells.

Advances in molecular tools and techniques will allow us to better understand the distribution of exchange and chromosome dynamics in both meiosis and mitosis.

## Figures and Tables

**Table 1:** Comparison between traditional screening tests and NIPT for chromosomal abnormalities.

Traditional Screening	NIPT
First and/or second trimester	Available from the 11th week of gestation
High DR when both first and second-trimester tests are performed and combined	Laboratory test performed only once
Ultrasound is a component of many algorithms	No ultrasound required
Includes screening for neural tube defects (second trimester)	No screening for neural tube defects
Rare indeterminate result	Approximately 1% inconclusive result
Decreased DR in twin pregnancies	DR in twin pregnancies equivalent to single pregnancies
Non-specific screening for CNVs	Targeted or genome-wide screening for CNVs available

CNV: copy number variation. DR: detection rate.

**Table 2:** Complete blood count (CBC) and hemoglobin electrophoresis (Hb A1 and Hb A2) of both consultands.

Analyte	CBC of the Father	CBC of the Mother	*Normal Range*
**RBC**	4.72 × 10^12^/L	3.56 × 10^12^/L	*4.5–5.7 × 10^12^/L*
**WBC**	5.5 × 10^9^/L	5.2 × 10^9^/L	*4.0–10.0 × 10^9^/L*
**HGB**	140 g/L	111 g/L	*133–167 g/L*
**HCT**	0.41	0.33	*0.35–0.53*
**MCV**	87 fL	94 fL	*77–98 fL*
**MCH**	29 pg	31 pg	*26–33 pg*
**MCHC**	33 g/L	33 g/L	*330–370 g/L*
**RDW**	14%	13%	*10.3–15.3%*
**Hb A1**	97.2%	97.5%	*96*.*5–98*.*5%*
Hb A2 *	2.8%	2.5%	*2*.*0–3*.*2%*

* HbF values between 0.1 and 0.4% are typically omitted from reporting, as they do not offer clinically relevant diagnostic insights [27].

## Data Availability

The original contributions presented in the study are included in the article, further inquiries can be directed to the corresponding author.
